# Fracture resistance and marginal fit of three different overlay designs using advanced zirconia-reinforced lithium disilicate CAD/CAM material

**DOI:** 10.1186/s12903-024-05315-1

**Published:** 2025-01-04

**Authors:** Heidi Saad Refaey, Sanaa H. Abdelkader, Yasser M. Aly

**Affiliations:** Department of Conservative Dentistry, Faculty of Dentistry, Alexandria University, Alexandria, Egypt

**Keywords:** Ceramics, Overlay, Marginal fit, Fracture resistance, CAD-CAM materials, Advanced lithium disilicate, Glass ceramics, Lithium disilicate

## Abstract

**Background:**

Conservative dentistry introduced modern restoration designs, contributing to the greater use of partial-coverage ceramic restorations. New strong bondable ceramic materials made fabricating partial coverage ceramic restorations easier to restore the badly destructed teeth.

**Aim of the study:**

This study investigated the impact of three distinct overlay preparation designs on the marginal fit (both before and after thermal aging) and the fracture resistance of overlay restorations fabricated using advanced zirconia-reinforced lithium disilicate (ALD) CAD/CAM glass-ceramic blocks.

**Materials and methods:**

Using a standardized preparation protocol, three typodont molars were prepared to receive three different indirect overlay ceramic restoration designs. The typodont teeth were duplicated to get 27 resin dies that were randomly allocated into three groups (*n* = 9) based on the preparation design; group (O): a traditional overlay preparation with anatomical occlusal reduction, group (OS): anatomical occlusal reduction with circumferential shoulder finish line, and group (OG): anatomical occlusal reduction with a central groove preparation at the mid-occlusal surface. After standardized restorations fabricated following the manufacturer’s guidelines, the restorations were cemented to their corresponding dies and exposed to thermal aging corresponding to 6-month clinical service. Marginal gap was measured before and after thermal aging procedure using an optical microscope. To measure fracture resistance, specimens were loaded till failure using the universal testing machine. The Kruskal Wallis test was utilized to assess data among the groups, followed by Dunn’s post hoc test with Bonferroni correction. Differences in the marginal fit before and after thermal aging were evaluated using Wilcoxon Sign Rank test.

**Results:**

A statistically significant difference in marginal fit was observed between the studied groups, with a p-value of 0.032 where group OS has the lowest micro gap compared to group OG and group O. The fracture resistance group (O) recorded the highest fracture resistance with a statistically significant difference between the studied groups at p value = 0.043.

**Conclusions:**

Adjusting the tooth preparation significantly influenced both the fracture resistance load and the marginal fit observed for advanced zirconia-reinforced lithium disilicate glass-ceramic (ALD) overlays.

## Background

There’s an increasing demand for minimally invasive and aesthetically pleasing tooth restorations, this explains the increasing popularity of partial-coverage ceramic restorations, as they offer a conservative approach to restore destructed tooth structure while achieving both cosmetic and functional demands [1, 2].

Adhesive dentistry introduced innovative restoration designs, resulting in a greater adoption of partial-coverage ceramic restorations. Overlay restorations have become a prevalent treatment option for posterior teeth with coronal damage. either due to caries or non-caries issues as abfraction [3–6].

Ceramic restorations merge between excellent biocompatibility and optimal optical and material properties, satisfying both patient and clinical demands [7–9], which is indicated for veneers, inlays, onlays, crowns, and FPDs [7–11].

Glass-ceramics, including lithium disilicate and zirconia-reinforced lithium silicate, are aesthetically appealing restorative materials fabricated with computer-aided design (CAD) computer-aided manufacturing (CAM) technologies. They were designed to replace metal-alloy frameworks, providing benefits in optical, physical, and biological properties [12, 13].

The durability of ceramic restorations primarily depends on the quality of bonding and the proper application technique. Cementation enhances fracture resistance by filling the irregularities on the etched fitting surface of the restoration, thereby enhancing bonding strength and preventing crack propagation especially the glass ceramics [14].

Maintaining fracture resistance is essential for ensuring the effectiveness of ceramic restorations. It is influenced by preparation design, ceramic material properties, restoration thickness, cementation technique, functional load, and internal ceramic defects [15]. Preparation designs, including cavity depth, width of the isthmus, the degree of taper, and the internal line angles, can influence fracture resistance. Additionally, the aging of the ceramic restoration/tooth complex may affect the failure rate [16].

The marginal fit of restoration is a critical element that affects the longevity of overlay restorations. Poor adaptation can create gaps between the restoration and tooth, leading to microleakage, plaque buildup, and potential recurrence of decay [13, 17, 18].

It’s worth mentioning that only a limited number of studies have investigated the impact of the design of preparation on the characteristics of overlay restorations, with most of these focusing mainly on the fracture resistance of the restorations [1, 3, 19–22]. Most of the previous pertinent research has assessed inlays and/or onlays that provide partial cusp coverage [4].

An information gap exists concerning the impact of preparation configuration on the marginal fit of ceramic overlays. Therefore, this research aimed to examine the marginal gap and fracture resistance of Advanced Zirconia-reinforced Lithium disilicate (ALD) overlays with various preparation designs. The null hypothesis for this study stated that there is no significant difference in marginal fit (before or after thermal aging) or fracture resistance among the studied groups.

## Materials and methods

### Study design and sample size

The study followed an in-vitro, parallel-controlled design, in which three parallel groups’ marginal fit and fracture resistance were evaluated. It was conducted in the laboratory of the Conservative Dentistry Department at the Faculty of Dentistry, Alexandria University, Egypt.

The sample size was estimated assuming a 5% alpha error and 80% study power. Based on the difference between independent means of previous study [4], the minimum sample size was calculated to be 7 samples per group, increased to 9 samples to make up for processing errors. Total sample = number per group x number of groups = 9 × 3 = 27 samples.

The sample size was calculated by G*Power 3.1.9.7.

### Specimen preparation

Three second mandibular molars typodont teeth (Columbia Dentoform Corporation, USA), were prepared using the following designs [4] :

- Group (O) a traditional overlay preparation with anatomical occlusal reduction 1.5 mm.
- Group (OS) an overlay preparation with anatomical occlusal reduction 1.5 mm and 1.0 mm shoulder finish line circumferentially.
- Group (OG) an overlay with anatomical occlusal reduction 1.5 mm and central groove preparation with a pulpal depth of 1.0 mm and a width of 2.0 mm.

The typodont teeth that had been prepared were subjected to scanning using an extraoral scanner (InEos X5; Dentsply Sirona, USA), to design three dies corresponding to the tooth preparations. Print out of twenty-seven dies made from 3D printing resin (Model Resin, Formlabs, Somerville, MA), which possess a comparable modulus of elasticity to dentin (modulus = 10 GPa). A laboratory printer (FormLab 2, Formlabs) was used [3, 23]. Each resin die was subsequently embedded in a copper metallic mold filled with auto-polymerizing acrylic resin (Acrostone, Egypt), exposing only the crown and 2 mm apical to the cementoenamel junction, simulating the bone level.

### Design and fabrication of overlay restorations

**Fig. 1:**
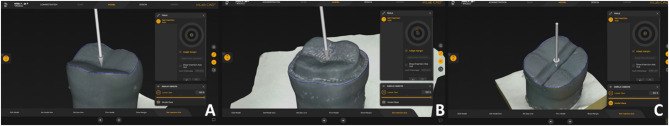
Shows the restoration design created using CAD technology on three distinct preparation designs: (**A**) group O, (**B**) group OS, and (**C**) group OG

A computer-aided design (CAD) digital software (inLab CAD SW 22.0.0; Dentsply Sirona) was used to design the restorations with a standardized morphology [Fig. 1] [3]. Twenty-seven overlays were fabricated using CAM technology (inLab MCXL, Dentsply Sirona) using Advanced Zirconia-reinforced lithium disilicate CAD/CAM blocks (Dentsply Sirona, Tessera ™).

### Grouping of specimens

Twenty-seven specimens were grouped according to the overlay design into three groups, nine for each. Overlay ceramic restorations were glazed and sintered based on the manufacturer’s guidelines.

### Cementation of the specimens

A 9.5% hydrofluoric acid (Porcelain Etch^®^, Bisco, USA) was applied on the dry intaglio surface of the restorations for 20 s, rinsed with a copious amount of water for 60 s, and air dried. Silane (Porcelain Primer; Bisco, Schaumburg, IL, USA) was applied on the etched restorations surface and air dried after 60 s. The overlays were then cemented to the prepared dies with a dual-cure resin cement following the manufacturer’s instructions (BisCem; Bisco, Schaumburg, IL, USA). Cement was auto-mixed and applied on both the intaglio surface of the restoration, and the prepared die surface, each restoration loaded with cement was gently seated on its corresponding prepared die, initially with finger pressure followed by application of a constant static load of 200 g [3, 23], by a specially designed device to ensure seating of the restoration for 60 s before photopolymerization. Then specimens were tack-cured for 2 s (Elipar 2500, 3 M Oral Care, St. Paul, MN) and excess cement was eliminated using scalpel, subsequently, a light curing procedure was applied for 20 s on each surface. Finally, the tooth-restoration interface was polished with polishing disks. The specimens were stored in water at room temperature before being tested.

### Tests

#### Marginal fit evaluation before thermal aging

**Fig. 2:**
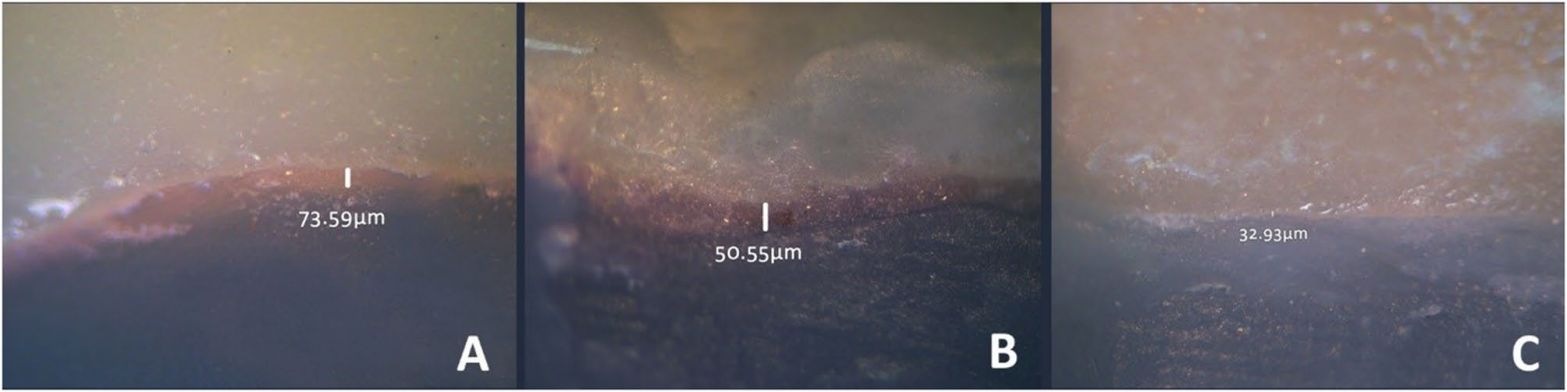
Marginal fit measurements before thermal aging for the three groups: (**A**) Group O, (**B**) Group OG, and (**C**) Group OS. Group OS (**C**) shows the least marginal gap before thermal aging

Evaluation of marginal fit was conducted following the methodology outlined by Holmes et al., [24] The fit accuracy of the overlays on their corresponding prepared teeth was measured twice before and after the thermal aging procedure. Six measurements were taken for each: mesiobuccal, midbuccal, distobuccal, mesiolingual, midlingual, and distolingual. The measurements were performed at a magnification of X20 and X110 using a stereomicroscope (Olympus SZ-1145TR Stereo Zoom Microscope; OLYMPUS Co) connected to a digital camera **[**Fig. 2**]**. The measurements were determined, and a mean value was calculated and performed by a single-blind examiner for each overlay.

#### Thermal aging

Thermal aging was performed with a custom-built device for a total of 5000 cycles representing a 6-month clinical service, between 5 and 55 degrees Celsius in water baths with dwell times of 15 Sects. [18, 19]. 

#### Marginal fit evaluation after thermal aging

**Fig. 3:**
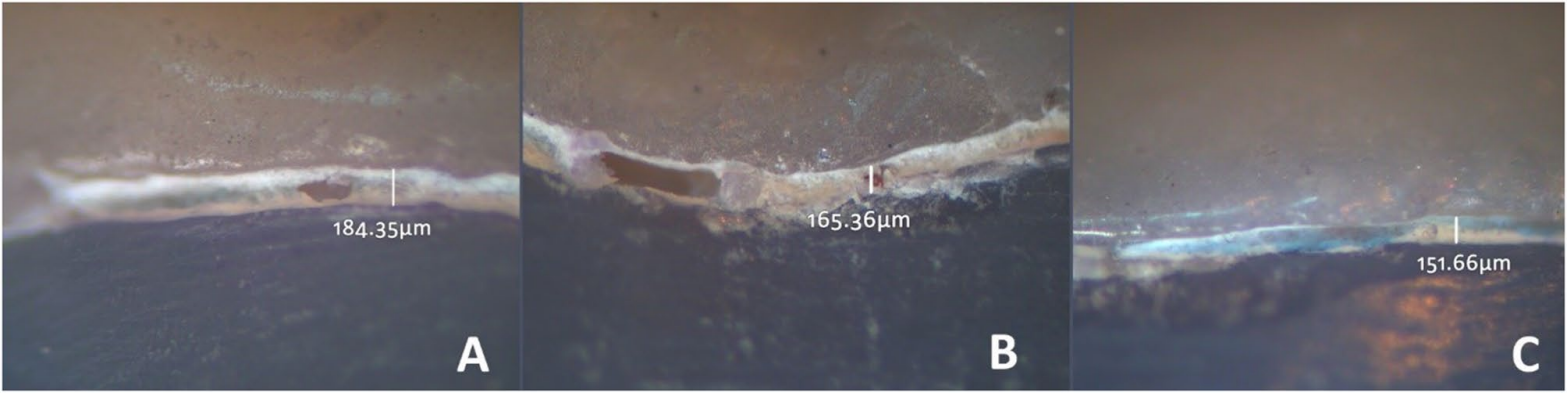
Marginal fit measurements after thermal aging for the three groups: (**A**) Group O, (**B**) Group OG, and (**C**) Group OS. The marginal gap increased after thermal aging in all three groups following the same pattern as before thermal aging where the maximum marginal discrepancy was noted in (**A**) group O

Following thermal aging, the measurements were assessed using a stereomicroscope at the same magnification and predefined measurement points [Fig. 3]. The collected data were then analyzed statistically after processing.

#### fracture resistance test

**Fig. 4:**
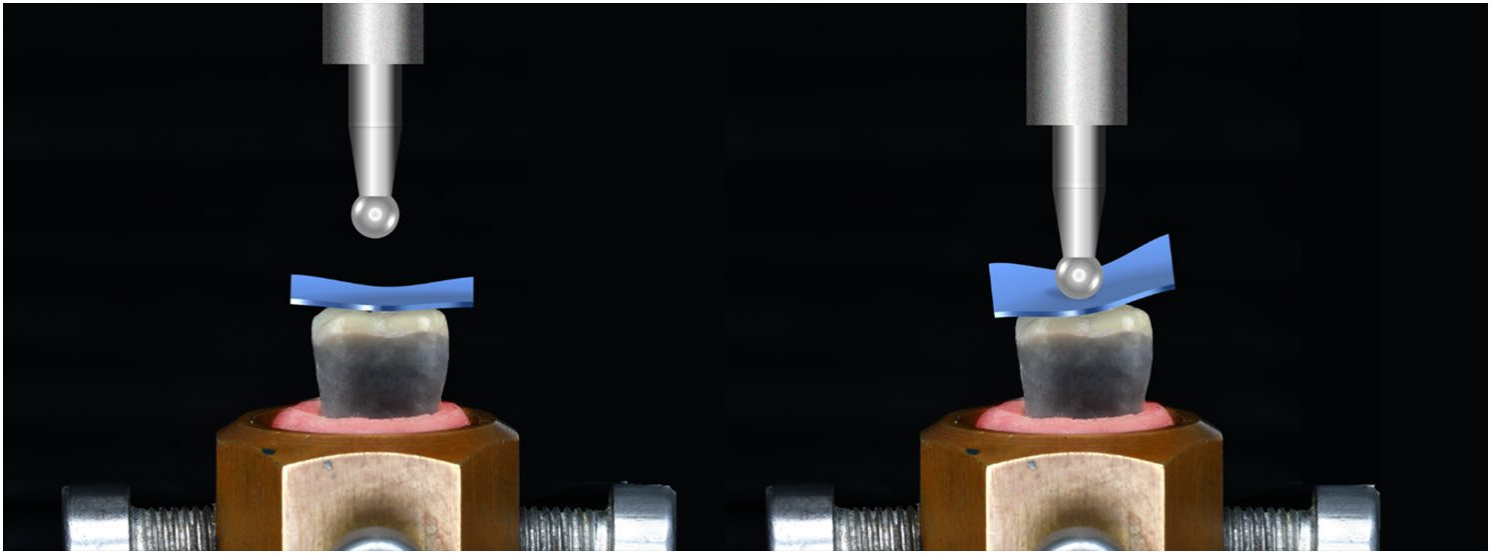
Schematic representation of the fracture resistance test setup. A universal testing machine (5ST, Tinius Olsen, England) applied load using a custom-made 6 mm diameter stainless-steel ball on the central fossa of the overlay along the long axis. A rubber sheet was placed beneath the sphere indenter to cushion and evenly distribute forces across the occlusal surface. The load was applied at a crosshead speed of 1 mm/min until fracture occurred

A universal testing machine (5ST, Tinius oslen, England) was used to evaluate the fracture resistance of the specimens. A stylus with a 6 mm diameter custom-made stainless-steel ball was used to apply the load on the central fossa on the occlusal surface of the overlay along the long axis. A rubber sheet was placed under the sphere indenter to serve as a cushion and distribute forces evenly at the occlusal surface. The crosshead speed was 1 mm/min until fracture [Fig. 4]. The software program (version 10.2.4.0; Horizon) automatically recorded the maximum loads for each specimen in newtons (N). The failure mode for each specimen was classified according to the structures involved in the fracture according to Burke’s classification (Table 1) [25].

**Table 1:** illustrates Burke’s classification of failure modes

Mode of failure	
I	Slight crack or fracture in restoration
II	Under 50% of the restoration lost
III	Fracture of the restoration extending through the midline
IV	Over 50% of the restoration lost
V	Serious fracture of the tooth structure and\or restoration

### Statistical analysis

Normality was checked using Shapiro Wilk test and Q-Q plots. Marginal fit and fracture resistance were not normally distributed thus both were presented mainly using median, minimum, maximum in addition to mean and standard deviation. The Kruskal Wallis test was used to analyze data between groups, followed by Dunn’s post hoc test with Bonferroni correction. Differences in marginal fit before and after thermal aging were analyzed using Wilcoxon Sign Rank test. All tests were two-tailed, with the significance level established at a p-value of ≤ 0.05. Data was analyzed using IBM SPSS, version 23 for Windows, Armonk, NY, USA.

## Results

### Marginal gap before and after thermal aging

**Fig. 5:**
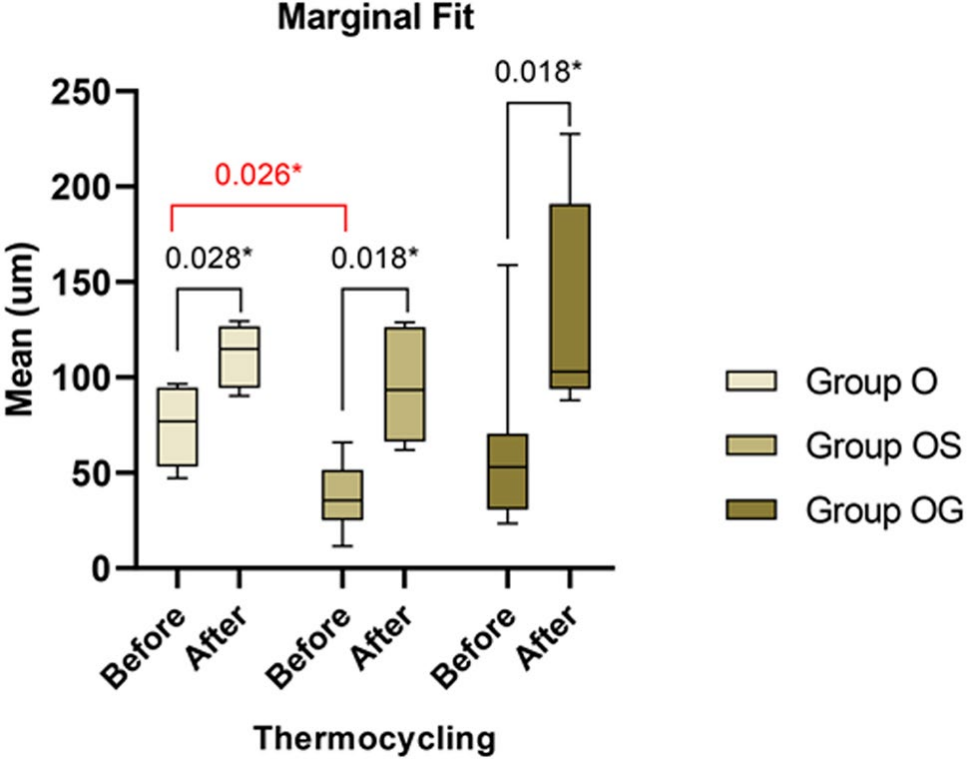
Waterfall Chart showing comparison of marginal fit results of the three studied groups before and after thermal aging. With a significantly increased marginal gap after thermal aging in all three groups following the same pattern as before the thermal aging

For marginal gap, the greatest mean marginal gap prior to thermal aging was observed in group O with a median gap value of 76.97 μm followed by group OG with a median gap value of 52.94 μm and the group OS with a median gap value of 35.57 μm. The marginal gap significantly increased after thermal aging in all three groups (*p* < 0.05) following the same pattern as before thermal aging where the maximum marginal discrepancy was noted in group O with a median gap 114.84 μm succeeded by group OG with a median gap value 103.01 μm and the minimum marginal gap was noted in group OS with a median gap value 93.50 μm [Fig. 5].

### Fracture resistance

**Fig. 6:**
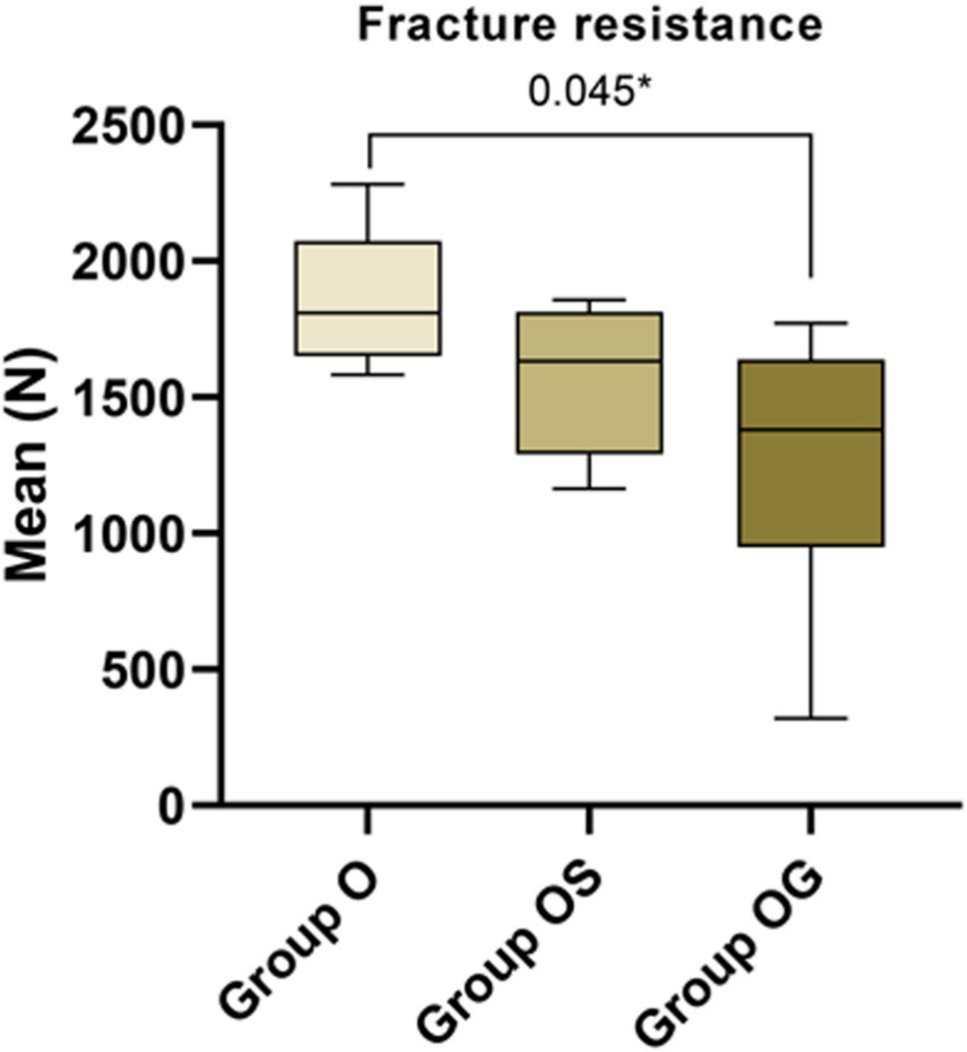
Waterfall chart showing a comparison of fracture resistance of the three studied groups. Group O had the highest median fracture load value of 1809.08 N, followed by Group OS, with a median fracture load value of 1632.77 N. The OG Group had the lowest median fracture load value, 1379.63 N

For fracture resistance, the O group had the highest median fracture load value 1809.08 N followed by Os group with median 1632.77 N. The OG group had the lowest median fracture load values of 1379.63 N [Fig. 6].

### Modes of failure

**Fig. 7:**
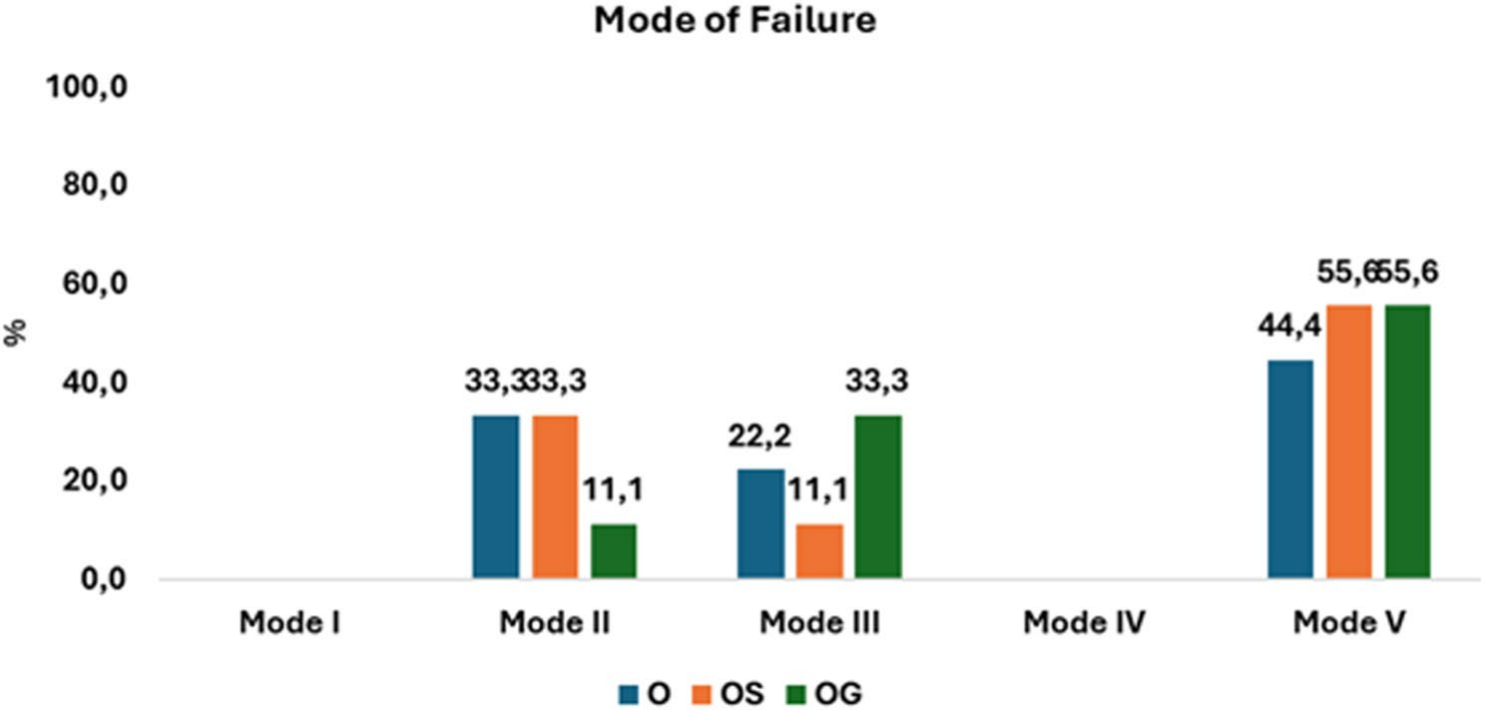
Chart showing a comparison of modes of fracture with no statistically significant difference between the three studied groups (*p* = 0.027)

**Fig. 8:**
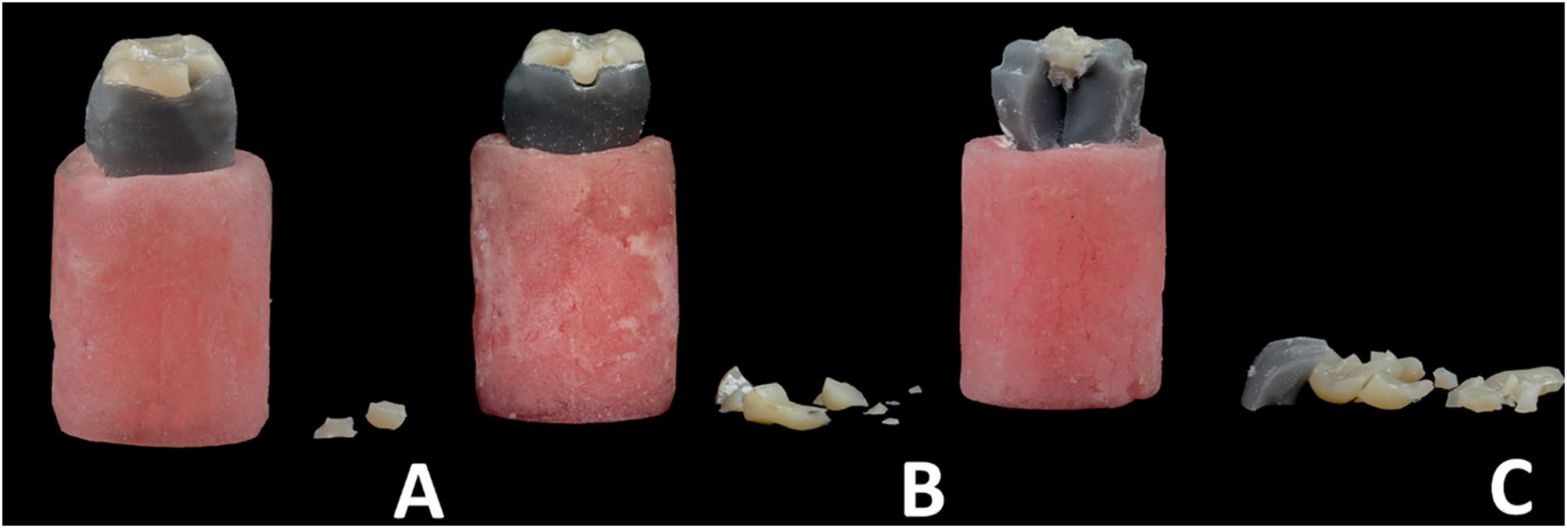
Illustrates the fracture mode observed in this study; as (**A**) mode II: less than half of the restoration lost. (**B**) mode III: Fracture of the restoration extending through the midline; half of the restoration lost. (**C**) mode V: Serious fracture of the tooth structure and restoration

**Fig. 9:**
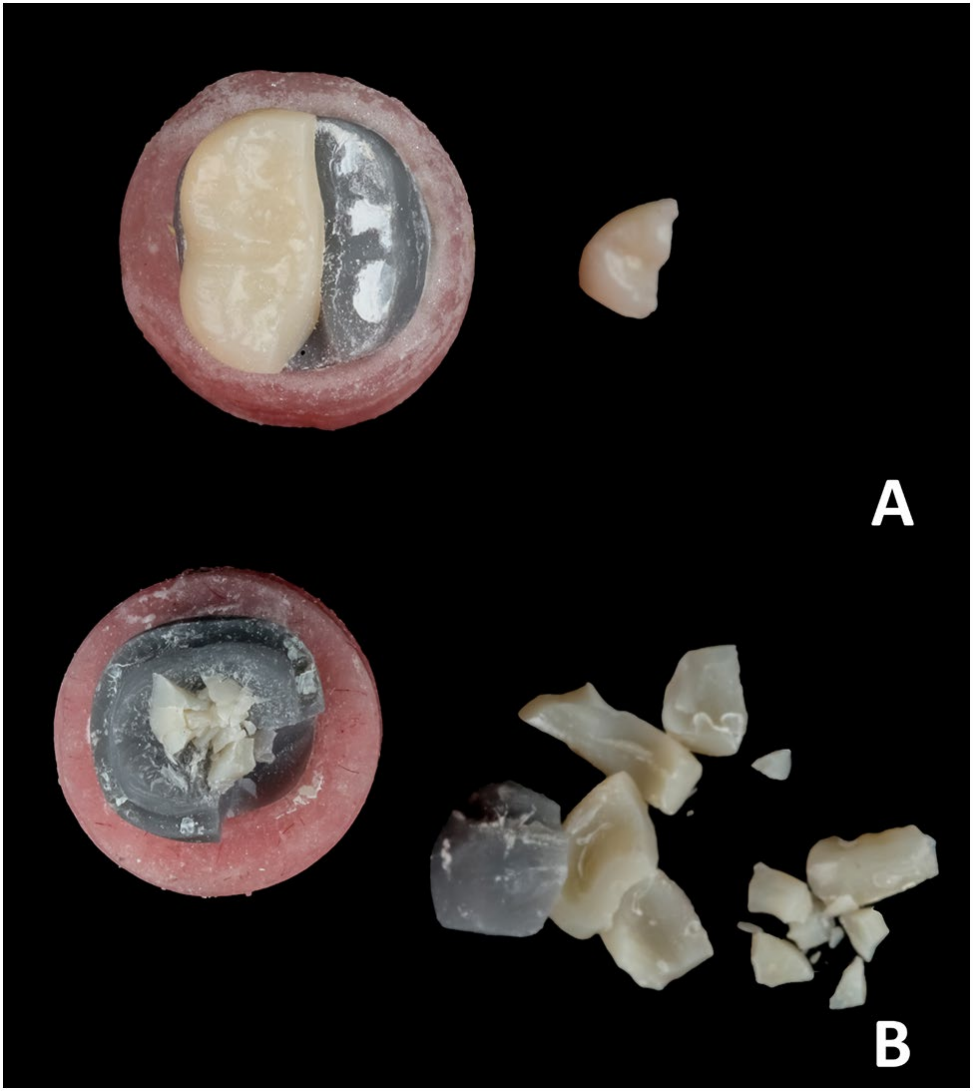
Presents the fracture modes from the occlusal view: (**A**) mode III: Fracture of the restoration extending through the midline and (**B**) mode V: Serious fracture of the tooth structure and restoration

Among the fracture modes [25], there is no statistically significant difference between the three studied groups (*p* = 0.027). In the (O) group (*n* = 9), 3/9 (33.3%) had mode II of fracture, 2/9 (22.2%) had mode III of fracture, and 4/9 (44.4%) had mode V of fracture. In group (OS) (*n* = 9), 3/9 (33.3%) had mode II of fracture, 1/9 (11.1%) had mode III of fracture, and 5/9 (55.6%) had mode V of fracture. In group (OG) (*n* = 9), 1/9 (11.1%) had mode II of fracture, 3/9 (33.3%) had mode III of fracture, and 5/9 (55.6%) had mode V of fracture, **[**Figs. 7 and 8, and 9**]** indicates the frequency of various modes of failure within the groups.

## Discussion

This research sought to evaluate and compare the marginal fit and fracture resistance of three different overlay designs using advanced zirconia-reinforced lithium disilicate CAD/CAM material. Both null hypotheses were rejected, as the preparation designs used significantly affected fracture resistance and marginal fit of studied groups. This outcome is consistent with prior research findings that demonstrated a substantial influence of preparation design on the marginal fit and the fracture resistance of restorations [4]. In this study, the marginal fit and fracture resistance of the advanced zirconia-reinforced lithium disilicate (ALD) CAD/ CAM material for three different overlay preparation designs on molars were compared.

This study selected advanced zirconia-reinforced lithium disilicate (ALD) over other materials, such as 5Y-Zirconia and novel fully crystallized lithium disilicate, due to its superior combination of mechanical strength and aesthetic properties. While 5Y-Z offers enhanced translucency, its reduced fracture toughness and absence of transformation toughening make it more prone to failure under stress. ALD, by contrast, provides a favorable balance between strength and translucency, making it particularly suitable for posterior restorations. Moreover, novel fully crystallized lithium disilicate materials were not fully stabilized or commercially available when the study was initiated. ALD also simplifies bonding protocols by eliminating the need for sandblasting, which can compromise surface integrity, thereby ensuring both durability and efficiency [23, 26]. Diligent efforts were made to replicate the clinical intraoral environment in this study. A single scanner system was used to standardize the intraoral scanning process, minimizing variations and ensuring more accurate results. Each preparation design was duplicated to produce standardized dies using 3D printing resin (Model Resin, Formlabs, Somerville, MA) possessing an elasticity modulus similar to that of dentin [3], for evaluation of ceramic overlays’ marginal adaptation and fracture resistance. The stimulation of the clinical environment was also taken into account in various aspects of the research, including tooth preparation, impression creation, procedure for fabrication, and cementation of restorations.

In this research, 3D-printed resin dies (Model Resin, Formlabs) were chosen over natural teeth due to their ability to provide consistent geometry across all specimens, eliminating variations in size and preparation design inherent to natural teeth. This approach also minimizes the challenges and variability associated with collecting, storing, hand-prepping, and handling natural teeth. Resin dies have a tensile strength of 61.0 MPa, which falls within the range of dentin tensile strength (44.4–97.8 MPa), making them a suitable substitute [3]. Studies have demonstrated that crowns cemented to resin die yield fracture strength results comparable to those cemented on natural teeth. For instance, one study concluded that resin dies closely mimic the fracture behavior of crowns on dentin. Furthermore, another study reported that zirconia crowns fractured on resin dies exhibited similar strength values to those fractured on enamel dies [3, 26]. They did not replicate the natural tooth structure for cement adhesion. However, adhesion was not a primary focus of our research.

Based on Holmes et al., [24] the term “marginal gap” denotes the vertical gap between the cervical edge of restoration and the prepared tooth surface. In simpler terms, the marginal gap pertains to the surface area of the cement, which is subjected to the oral environment and is susceptible to degradation [24]. In this study the marginal gap was evaluated using the direct-view technique using a stereomicroscope. Some earlier research has also utilized this method [27, 28].

The marginal fit was evaluated in this study before and after thermal aging. The findings indicated that the marginal gap for all groups was below 120 μm before and after thermal aging, falling within the range considered clinically acceptable as determined by the Consensus among most authors suggests [13, 29]. Comparing data on marginal adaptation from various studies poses challenges and potential inaccuracies due to several factors, including variations in preparation design, measurement techniques, the number and location of measurement points, the type of resin cement employed, and the method used to fabricate the restoration. Advanced 4- or 5-axis milling machines, which can enhance the fit between the prepared tooth structure and the restoration’s intaglio surface, also contribute to these variations. However, the precision and reliability of the outcomes improve when more measurement points are included and consistently assessed at the same locations across samples. This approach reduces variability, ensuring that the results accurately reflect the restoration’s adaptation quality. Our study adopted this approach to ensure accurate and reliable results. Therefore, carefully considering these variations is essential when making in vitro comparisons of data [5, 9, 15, 30]. In this research, the restorations were adhered under 200 g applied weight to standardize the pressure applied [3, 23]. However, to ensure consistency in the cementation procedure, all restorations were placed by the same operator. Based on the findings of this research, group OS revealed the minimal marginal gap after cementation with a median gap value of 35.57 μm, succeeded by group OG with a median gap value of 52.94 μm and group O with a median gap value of 76.97 μm.

Therefore, the preparation design of group OS (overlay preparation with 1.5 mm anatomical reduction of the occlusal surface and 1.0 mm circumferential shoulder finish line) has more retentive form and more defined margins, resulting in a reduction of the marginal gap size. These results aligned with the findings of Yang et al., [5] who assessed how tooth preparation design influences the marginal adaptation of composite resin CAD-CAM Onlays by comparing two different preparation designs. They noted that the conventional design, which provided more excellent retention, resulted in greater adaptation. However, this result contradicted the findings of Falahchai et al., [4] and Kim et al., [6] Who determined that preparation designs lacking retention would offer superior adaptation to those with retention features. The variation can be ascribed to the different materials employed for restoration fabrication, variations in preparation designs, cementation techniques, and distinct techniques for gap measurement.

In this research, there was a reduction in marginal fit (increase in micro gap) in all groups after exposure to thermal aging compared to the initial measurements. This observation aligned with the outcomes reported in earlier studies [9, 18]. They reported that the thermal aging affected all groups’ marginal fit and showed decreasing marginal adaptation. This finding was in agreement with the outcomes obtained in the current study. In group O, the statistical significance of the change in marginal gaps was noticeably lower compared to group OG and group OS. This may be due to the nature of 3D printing resin material; the temperature variations cause the resin to undergo expansion and contraction, particularly in thin margins [18].

In the current study, the fracture resistance was evaluated for all groups after thermal aging. The force required to cause a fracture in the group (O) was 1809.08 N. However, it was 1632.77 N for group (OS). The lower value was recorded in group (OG) 1379.63 N. Studies showed that the occlusal forces generated during chewing and biting usually reach approximately 100 N, with a maximum bite force in habitual occlusion of up to 320 N [23]. These values are considerably lower than the fracture resistance observed in our results, suggesting that partial restorations can comfortably endure occlusal forces without risk of failure. The durability of all-ceramic restorations is impacted by various factors, such as the ceramic material’s microstructure and fatigue, fabrication methods, preparation design, and bonding technique [16, 20]. The results of this study showed that group O (teeth with 1.5 mm anatomical occlusal reduction that received occlusal veneers) and group OS (teeth with 1.5 mm anatomical occlusal reduction and 1.0 mm circumferential shoulder finish line preparation) exhibited the highest fracture resistance of the studied groups under the imposed forces (1809.08 N and 1632.77 N), This observation was consistent with the results obtained from Clausen et al., [20] Who evaluated occlusal overlays and the influencing of ceramic material and preparation design. They reported that all-ceramic full coverage restorations bonded exclusively to enamel exhibited a tendency towards increased fracture resistance compared to those bonded to dentin with a finishing line located in enamel. Falahchai et al. [1] revealed that teeth with less extensive restorations and sound marginal ridges demonstrated a lower incidence of fractures, reporting that increasing the reduction of tooth structure in the central area, such as with a mesio-occluso-distal preparation, leads to compromising the strength of the remaining tooth structure. Alternatively, the intracoronal extension of an overlay may produce a wedging effect. Hence, this clarifies why the fracture resistance outcomes of group (OS) exceeded those of group (OG) (characterized by anatomical occlusal reduction with a central groove), where both the central portion of the tooth and marginal ridges were eliminated. Also, preparation designs that emphasize retention have complex shapes with sharp inner edges, making them more prone to developing predetermined weak points. These geometric changes can lead to localized stress concentrations [1]. This finding aligned with Channarong et al. [19], Who evaluated the resistance of bonded ceramic overlay restorations to fractures as influenced by different preparation designs. They found that the more natural tooth structure that remains, the more favorable the long-term prognosis for that tooth. Therefore, the conservation of natural tooth structure is crucial for the overall lifespan of the tooth. Also, they demonstrated that overlay restorations utilizing the adhesive system could reinforce defective teeth to a fracture resistance level similar to that of intact teeth. In contrast, the margin type and axial wall length did not affect the final fracture resistance. However, these results contradicted Alberto et al.‘s findings [3]. They reported that the fracture resistance of the full-coverage crown restorations was notably greater than that of the occlusal-cover restorations. Also reported that the effect of the amount of the preparation that was 2.0 mm and 1.5 mm thick needed significantly more fatigue cycles to fail (17 − 15 times fatigue cycles) compared to crowns with a thickness of 1.0 mm. Also, the study has shown that self-adhesive resin cement exhibits weaker mechanical and bonding properties compared to conventional resin-luting cement [3]. However, previous research has demonstrated that a 1 mm thin layer of lithium disilicate ceramic bonded to enamel exhibits a lower risk of fracture compared to bulker restorations (1.5–2 mm as recommended) bonded to dentin, primarily because of reduced mechanical complications [19]. This confirms that other factors like ceramic thickness and type, type of cementation, tooth architecture, and study design can influence fracture resistance in addition to preparation design. Previous research indicated that any loss of tooth structure, whether caused by caries or cavity preparation, reduces the fracture resistance of restorations. The literature suggests that more conservative preparation designs with lower retention features tend to enhance fracture resistance [4]. Building on the previous findings, this explains the results observed in our study. Group O, which featured the most conservative preparation design with no retentive form, demonstrated the highest fracture resistance. In contrast, Group OG, which included a central groove and internal angles, showed increased stress concentration in these areas, leading to reduced fracture resistance.

According to the study’s results, various preparation designs exhibited distinct. frequencies of modes of failure. Nevertheless, these differences were not significant. More than half of the restoration fractures noted in this study involved damage to both the die and the restorative material. This observation is consistent with the results documented in previous studies by Johnson et al., [21] And Alberto et al., [3].

This study had several limitations. These limitations include that it was conducted in vitro, differing from a clinical setting where factors such as saliva and restricted access for the scanner inside the oral cavity would affect the precision of scanning prepared teeth. The internal adaptability was not evaluated in the study and faced challenges in accurately simulating a clinical setting.

## Conclusions

According to the results of this research:

1. All tested groups showed marginal fit results within clinically acceptable parameters.
2. All preparation designs utilized in this study for creating overlay restorations from ALD ceramics demonstrated acceptable fracture resistance.
3. Higher fracture resistance was shown with intact marginal ridges and less extensive preparations.
4. Modification of tooth preparation significantly impacted the magnitude of the fracture resistance and the marginal gap observed around ALD overlays.
5. Thermal aging affected the marginal gaps of the three groups following the same pattern as before thermal aging.

## Data Availability

Data AvailabilityThe datasets created and analyzed during this study can be obtained from the corresponding author upon reasonable request.

## Abbreviations

ALD: Advanced lithium disilicate
CAD/CAM: Computer-aided design /computer-aided manufacturing
mm: Millimeter
no.: Number
µm: Micrometer
°C: Celsius or centigrade
N: Newton
SD: Standard deviation
